# Outcomes in patients with non‐invasive breast carcinoma

**DOI:** 10.1002/cnr2.1768

**Published:** 2022-12-09

**Authors:** Ryungsa Kim, Ami Kawai, Megumi Wakisaka, Mika Shimoyama, Naomi Yasuda, Mitsuya Ito, Takanori Kin, Koji Arihiro

**Affiliations:** ^1^ Department of Breast Surgery Hiroshima Mark Clinic Hiroshima Japan; ^2^ Department of Breast Surgery Hiroshima City Hospital Hiroshima Japan; ^3^ Department of Anatomical Pathology Hiroshima University Hospital Hiroshima Japan

**Keywords:** breast cancer, isolated tumor cell, non‐invasive carcinoma, recurrence, sentinel lymph node

## Abstract

**Background and Aim:**

Non‐invasive breast carcinoma is considered to be localized disease and is distinguished from invasive ductal and lobular carcinomas. The local recurrence of non‐invasive carcinoma after surgery may lead to development of invasive carcinoma and promote distant metastasis, which worsens the prognosis for breast cancer mortality. The distant metastasis of non‐invasive carcinoma may involve the ductal microvasculature without invasion. The outcomes of non‐invasive breast carcinoma were examined in this retrospective cohort study.

**Methods and Results:**

Of 872 primary breast cancers diagnosed at a single center between May 2008 and March 2022, 93 (10.6%) were found to be non‐invasive carcinomas and were examined in this study. The breast cancer recurrence and survival rates of patients with non‐invasive carcinoma were analyzed retrospectively. The median follow‐up period was 1891 (range, 5–4804) days. All patients underwent surgical treatment [mastectomy with sentinel lymph node biopsy (SLNB) and partial mastectomy with or without SLNB, tumorectomy, and microdochectomy]. Postoperatively, radiation therapy was administered to 73 (78.4%) of the patients and endocrine therapy was administered to 64 (81.0%) of 79 patients with hormone‐receptor positivity. Of 26 patients who underwent partial mastectomy with SLNB, 24 (92.3%) showed isolated tumor cells in the SLNs on one‐step nucleic acid amplification. Local recurrence was observed in three (0.3%) patients; no distant metastasis was observed. One patient died of a noncancerous disease. The overall survival rate was 98.0% and the breast cancer‐specific survival rate was 100.0%.

**Conclusions:**

Non‐invasive breast carcinoma, like invasive breast carcinoma, causes local recurrence, but has a good prognosis without distant metastasis. The clinical significance of isolated tumor cells in the SLNs as a systemic component of non‐invasive breast carcinoma remains to be elucidated.

## 1 INTRODUCTION

Breast cancer is classified histologically as invasive or non‐invasive based on whether tumor cells in the ductal component pass through the epithelial basement membrane.^1^ Invasive and non‐invasive carcinomas are also classified as ductal or lobular based on the confinement of cells and sites of origin to the ducts or lobules.^1^ Invasive ductal carcinoma (IDC) and non‐invasive ductal carcinoma of the breast [ductal carcinoma in situ (DCIS)] are more common than lobular carcinoma. Genetic and epigenetic events occurring in a single cell promote the transition of DCIS to invasive carcinoma and trigger metastatic spread to distant organs.^2^ In IDC with accumulated genetic abnormalities combined with clonal expansion and selection, tumor cells that have penetrated the epithelial basement membrane invade not only the vascular component, but also the stromal component and fat tissue, resulting in distant metastasis as systemic disease.^2^ Non‐invasive carcinomas, such as DCIS and lobular carcinoma in situ (LCIS), are considered to be local diseases and do not develop distant metastasis unless they progress to invasive carcinoma with local recurrence after surgical treatment.^3^

The sentinel lymph nodes (SLNs) in the axilla form the first immunological barrier to tumor metastasis; the evaluation of SLN metastasis is generally performed on frozen or permanent sections. One‐step nucleic acid amplification (OSNA)^4^, ^5^, ^6^ involves the amplification of cytokeratin (CK)19 mRNA from lysate for the determination of SLN status. It is considered to be the most accurate method for the intraoperative diagnosis of lymph node metastasis. A recent study revealed no difference in disease‐free survival or overall survival (OS) rates for patients with stages I and II breast cancer between those diagnosed using OSNA, frozen section analysis, and definitive histological analysis.^7^

Sentinel lymph node biopsy (SLNB) is a standard procedure in breast cancer surgery.^8^ It is used for the surgical treatment of DCIS with tumor formation or lesion extension due to the possibility of invasive carcinoma. The presence of tumor cells in the SLNs may be due to their iatrogenic dissemination into the lymphatic or vascular system via diagnostic biopsy [fine‐needle aspiration (FNA), core needle, excisional, or incisional]^9^, ^10^, ^11^ or to the microvascular invasion of DCIS, which may cause distant metastasis.^12^ However, the clinical significance of these phenomena, and whether the systemic extension of non‐invasive carcinoma is due to the development of invasive carcinoma or occurs via invasion of the breast ductal microvasculature, remain to be elucidated. Based on this background, we conducted a retrospective cohort study of breast cancer recurrence and survival in patients with non‐invasive carcinoma, exploring whether this carcinoma is a local or systemic disease.

## 2 METHODS

### 2.1 Patients and procedure overview

This retrospective study was conducted using data from women with non‐invasive carcinoma (International Union for Cancer Control TNM stage 0)^13^ from among the sample of all patients with primary breast cancer diagnosed at the Hiroshima Mark Clinic between May 2008 and March 2022. Patients with non‐invasive carcinoma with final pathological diagnoses made based on surgical specimens were included; patients with microinvasive or concurrent invasive cancer and those who had received neoadjuvant therapy were excluded. In addition, patients diagnosed with non‐invasive carcinoma based on preoperative vacuum‐assisted biopsy who were ultimately found to have invasive carcinoma based on surgical specimen analysis were excluded.

According to patients' preferences, outpatient surgery under local anesthesia (LA) and intravenous anesthesia (IVA) and/or sedation was performed at the clinic,^14^ and inpatient surgery or total mastectomy (Bt) under general anesthesia (GA) was performed at an associated hospital. Breast‐conserving surgery (BCS) was performed as an outpatient or inpatient procedure according to eligible patients' preferences, and was not mandatory. This cohort study was approved by the Ethics Committee of Hiroshima Mark Clinic (no. HMC‐04), and all treatments were performed with patients' informed consent.

### 2.2 Anesthesia

In outpatient surgeries performed during the study period, LA with lidocaine, benzodiazepines (e.g., diazepam and midazolam) as sedatives, IVA with propofol, and pentazocine (opioid receptor partial agonist) and pethidine (synthetic opioid) as analgesics were used. Preoperatively, 10 ml 0.5% lidocaine was injected locally under ultrasound guidance into the retromammary tumor space when marking the tumor resection site; additional local injections were administered around the tumor before and during surgery. For the intraoperative monitoring of biological functions, each patient was fitted with a biometric monitor to measure the pulse rate, electrocardiographic data, blood pressure, respiratory rate, and oxygen saturation. All patients received oxygen at a rate of 3–5 L/min delivered by oxygen mask during surgery, with preparation for emergency mask ventilation and tracheal intubation. In inpatient surgeries, GA was induced with total IVA using propofol and synthetic opioids such as remifentanil, or volatile anesthesia with sevoflurane using synthetic opioids.

### 2.3 Surgical procedures

The patients underwent SLNB and BCS with partial (Bp) or quadrant (Bq) breast resection or Bt. BCS was defined as resection of the primary tumor and partial mastectomy with a 1–1.5‐cm margin. In outpatient surgeries, axillary SLNB was performed using indigo carmine and indocyanine green dyes; in inpatient surgeries, it was performed using the radioisotope ^99m^Tc‐phytate and indigo carmine dye. During outpatient surgeries, OSNA assays^4^, ^5^ (Sysmex, Kobe, Japan) were performed to assess SLN metastasis via the quantification of CK19 mRNA copy numbers in homogenized SLN samples. OSNA results were classified as reflecting macrometastasis (2+; CK19 mRNA copy number ≥5.0 × 10^3^/μL), micrometastasis (1+; ≥ 2.5 × 10^2^/μL and <5.0 × 10^3^/μ), and negativity [−; elevated (with isolated tumor cells); < 0 and <2.5 × 10^2^/μL or flat (with no tumor cells); 0].^4^ Specimens from outpatient surgeries were sent to the Fukuyama Medical Laboratory Co., Ltd. (Fukuyama, Japan), where they were processed for pathological analysis and examined by a pathologist who made the final diagnoses. During inpatient surgeries, SLNs on frozen sections were evaluated; the findings were confirmed postoperatively using permanent sections.

### 2.4 Immunohistochemical assays

To evaluate the expression of hormone receptor (HR), estrogen receptor (ER), progesterone receptor (PR), and human epidermal growth factor receptor 2 (HER‐2) proteins, formalin‐embedded sections of surgical tumor specimens were immunostained using the BenchMark ULTRA automated immunohistochemical slide staining system (Roche Diagnostics, Basel, Switzerland) at Fukuyama Medical Laboratory Co., Ltd. The antibodies used were anti‐ER (SP‐1), anti‐PR (1E2), and anti–HER‐2 (4B5; all from Ventana, Yokohama, Japan). A pathologist graded ER and PR expression using the Allred score,^15^ calculated based on proportion and intensity, with scores ≥3 reflecting receptor positivity. HER‐2 expression was assessed using the American Society of Clinical Oncology/College of American Pathologists clinical practice guidelines,^16^ with positivity defined as strong complete membrane staining in >10% of tumor cells.

### 2.5 Local and systemic therapies

Patients who underwent BCS received postoperative radiotherapy (RT) for the remaining breast, delivered at the affiliated hospital at standard doses for 3 weeks (hypofractionated dose: 40.5 Gy/15 Fr) or 4 weeks (conventional dose: 50 Gy/25 Fr) after surgical treatment. Actual radiation doses were adjusted according to body surface areas. Patients with resection margins ≤5 mm or age ≤ 50 years were given RT boosters to the tumor bed (10.8 Gy/4 Fr or 10 Gy/5 Fr) as needed. Patients with HR positivity received endocrine therapy (ET) with tamoxifen (TAM, 20 mg) for 5 years.

### 2.6 Survival analysis

All data were analyzed using Satcel 4 software (OMS Publishing Inc., Tokyo, Japan). Cumulative OS and breast cancer‐specific survival (BCSS) rates and durations from the date of surgery were calculated using the Kaplan–Meier method. OS was defined as the time from surgical treatment to death from any cause, and BCSS was defined as the time from the date of surgical treatment to the date of death attributable to breast cancer.

## 3 RESULTS

### 3.1 Patients' clinicopathological characteristics

Of 872 patients with primary breast cancer diagnosed at our clinic, 94 (10.7%) patients were diagnosed with non‐invasive carcinoma. One patient did not receive surgical treatment after diagnosis and was excluded from this study. The clinicopathological characteristics of the remaining 93 patients are shown in Table 1. The sample comprised 87 cases of DCIS, two cases of LCIS, three cases of co‐existing DCIS and LCIS, and one case of non‐invasive apocrine carcinoma. The median age was 47 (range, 27–80) years. Sixty‐three patients underwent outpatient surgery at our clinic and 30 patients underwent inpatient surgery at the affiliated hospital. Seventy‐nine (84.9%) and 20 (21.5%) patients had HR‐positive and HER‐2‐positive tumors, respectively. Twenty‐two (23.6%), 46 (49.4%), and 23 (24.7%) tumors were of nuclear grades 1, 2, and 3, respectively. The types of preoperative diagnostic biopsy were FNA cytology (*n* = 78), core needle biopsy (CNB; *n* = 2), vacuum‐assisted biopsy (VAB; *n* = 54), and other (*n* = 1). The most common surgery performed was partial mastectomy (Bp/Bq) with SLNB [*n* = 59 (63.4%)], followed by Bp/Bq and Bt with SLNB [*n* = 15 (16.1%) each]. Of 26 patients who underwent SLNB for whom OSNA was performed, 24 (92.3%) showed elevated negativity with isolated tumor cells; VAB was performed in 13 of these 24 patients. ET was administered to 64 (68.8%) patients. Postoperative RT was administered to 73 (78.4%) patients, and 43 of these patients received RT boosters.

**TABLE 1:** Clinicopathological characteristics of 93 patients with non‐invasive carcinoma

Characteristic	No. of patients
Median age (range), years	47 (27–80)
Histology	
DCIS	87
LCIS	2
DCIS/LCIS	3
Non‐invasive apocrine carcinoma	1
Grade	
1	22
2	46
3	23
Unknown	2
HR	
Positive	79
Negative	11
Unknown	3
HER‐2	
Positive	20
Negative	70
Unknown	3
Surgical treatment	
Outpatient	63
Inpatient	30
Diagnostic biopsy	
FNA	78
CNB	2
VAB	54
Other	1
Surgical procedure	
Bt + SLNB	15
Bp(q)	15
Bp(q) + SLNB	59
OSNA	26
Elevated type	24
After VAB	13
No VAB	11
Flat type	2
After VAB	1
No VAB	1
Tm	1
MDT	3
Postoperative RT	
Yes	73
Boosted	43
No	17
Unknown	3
Postoperative ET	
Yes	64
No	24
Unknown	5

Abbreviations: Bp(q), partial or quadrant breast resection; Bt, total mastectomy; CNB, core needle biopsy; DCIS, ductal carcinoma in situ; ET, endocrine therapy; FNA, fine‐needle aspiration; HER‐2, human epidermal growth factor receptor 2; HR, hormone receptor; LCIS, lobular carcinoma in situ; MDT, microdochectomy; OSNA, one‐step nucleic acid amplification; RT, radiation therapy; SNLB, sentinel lymph‐node biopsy; Tm, tumorectomy; VAB, vacuum‐assisted biopsy.

### 3.2 Breast cancer recurrence and survival

During a median follow‐up period of 1891 (range, 5–4004) days, three patients experienced breast cancer recurrence (Table 2). One patient had local recurrence from DCIS to IDC and underwent Bt with SLNB. This patient had a HER‐2‐positive (3+) tumor and underwent postoperative RT with a booster. One patient had local recurrence from non‐invasive to invasive lobular carcinoma (ILC) and underwent repeat partial mastectomy. She had a HER‐2‐positive (3+), PR‐negative, HR‐negative tumor with ≤1% ER expression (Allred score = 2). This patient underwent ET with TAM and postoperative RT with a booster. HER‐2 targeted therapy was not used because the tumor diameter was <5 mm. The third patient had local recurrence of DCIS and underwent alternative rather than standard treatment, according to her preference; she had a HER‐2‐negative tumor and received ET with TAM, but no RT. The initial surgical margins were well over 2 mm (3 mm and 11 mm, respectively) in the first two cases; in the last case, the DCIS was exposed but the patient refused additional resection of the remaining breast.

**TABLE 2:** Clinicopathological characteristics of local recurrence in three patients with non‐invasive carcinoma

Patient no.	Age at diagnosis (years)	Histology	Surgical treatment/margin	Postoperative RT (booster)	Postoperative ET	DFI	Histology at time of LR	Treatment after LR	Prognosis
1	27	DCIS, G2, HR^−^/HER‐2^+^	Bp/3 mm	60 Gy/30 Fr (+)	None	2 Y 11 M	IDC, HR^−^/HER‐2^+^	Bt + SLNB, TC/Tz	9 Y 10 M, alive
2	47	LCIS, G3, HR^−^/HER‐2^+^	Bp + SLNB/11 mm	60 Gy/30 Fr (+)	TAM	4 Y	ILC, HR^−^/HER‐2^+^	Bp	7 Y 5 M, alive
3	31	DCIS, G2, HR^+^/HER‐2^−^	Bp/DCIS exposure	None	TAM	2 Y 4 M	DCIS	Alternative therapy	3 Y 11 M, alive

Abbreviations: Bp, partial breast resection; Bt, total mastectomy; DCIS, ductal carcinoma in situ; DFI, disease‐free interval; ET, endocrine therapy; G, grade; HER‐2, human epidermal growth factor receptor 2; HR, hormone receptor; IDC, invasive ductal carcinoma; ILC, invasive lobular carcinoma; LCIS, lobular carcinoma in situ; LR, local recurrence; M, months; RT, radiation therapy; SLNB, sentinel lymph‐node biopsy; TAM, tamoxifen; TC, docetaxel/cyclophosphamide; Tz, trastuzumab; Y, years.

**FIGURE 1:**
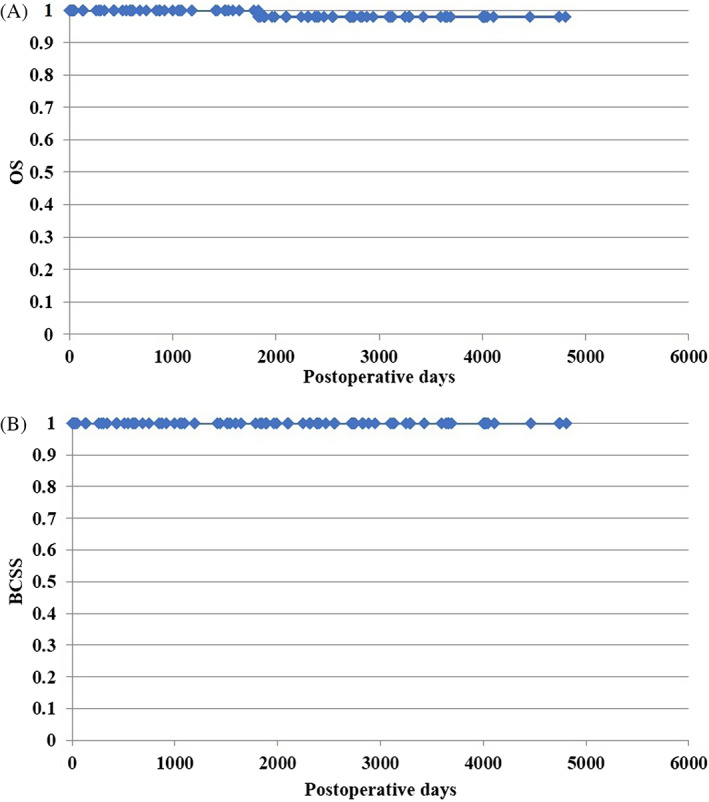
Cumulative overall survival (OS, A) and breast cancer‐specific survival (BCSS, B) for 93 patients with non‐invasive carcinoma

No breast cancer‐related deaths occurred, but one patient died following a stroke. The cumulative OS and BCSS rates for the entire cohort were 98.0% and 100%, respectively (Figure 1).

## 4 DISCUSSION

In the present cohort of 93 patients, no distant metastasis of DCIS or LCIS occurred after surgical therapy. Local recurrence was seen in three patients; two cases were salvaged by total and repeat partial mastectomies, and the third case was managed with alternative therapy. The two surgically‐treated cases were IDC and ILC with no distant metastasis at diagnosis. These findings suggest that local recurrence due to the transition from non‐invasive to invasive carcinoma is not a cause of distant metastasis associated with an increase in breast cancer mortality. The results of the present study are consistent with previous reports that the local recurrence of DCIS after lumpectomy is reduced by postoperative RT, possibly in combination with adjuvant ET with TAM, relative to lumpectomy alone.^17^, ^18^, ^19^ However, the presence of isolated tumor cells in the SLNs may suggest the possibility of metastasis without the invasive conversion of non‐invasive carcinoma, although no distant metastasis was observed in this study. Whether such isolated cells are involved in the development of distant metastasis in non‐invasive carcinoma cases remains to be determined.

The incidence of distant metastasis in patients with DCIS is low, but the reduction of local recurrence does not correlate with breast cancer mortality,^17^, ^20^, ^21^ suggesting that tumor cells can invade the breast duct microvasculature as systemic disease in these patients.^12^ Indeed, OSNA revealed isolated tumor cells in the SLNs in 24 of 26 patients in the present study. Thirteen of these 24 patients had undergone preoperative VAB. OSNA‐detected micrometastasis has been suggested to be due to the mechanical disruption of the tubular component during CNB for the histological evaluation of DCIS.^22^ Micrometastasis in DCIS has been proposed to be attributable to true metastasis due to occult invasion, and to iatrogenic dissemination by diagnostic CNB.^22^, ^23^ Repeat histopathological evaluation of surgical specimens has revealed occult invasion in some DCIS cases, but this does not account for about 40% of such cases.^23^ In another study, DCIS cases showed SLN positivity despite the absence of occult invasion on comprehensive histological sectioning.^24^ Because the accidental transfer of tumor cells to the SLNs by FNA and CNB between biopsy and surgical treatment is less frequent than SLN metastasis,^25^, ^26^ the iatrogenic seeding of isolated tumor cells and micrometastasis in the SLNs are not likely to be caused by diagnostic biopsy prior to surgical treatment.

In support of this point, the incidence of isolated tumor cells in SLNs was the same in patients who did and did not undergo VAB in this study. In addition, 16%–44% of circulating tumor cells in patients with DCIS have been reported in the peripheral blood and bone marrow, with no difference in their frequency from that in patients with IDC.^27^, ^28^, ^29^ These findings suggest that circulating tumor cells in patients with DCIS enter the circulating blood before passing through the epithelial basement membrane.^12^

SLNB is no longer recommended for patients diagnosed with DCIS by CNB and treated with BCS.^30^ SLNB can be performed even after the final pathological diagnosis of invasive breast carcinoma in surgical specimens, as SLN metastasis occurs in only 2% of cases with the final diagnosis of pure DCIS.^30^ This approach has the advantage of eliminating the risk of complications and reducing medical costs. SLNB can be performed safely with no serious complications, and in the presence of extensive DCIS, a palpable mass, high‐grade DCIS, or comedo necrosis, its performance can avoid subsequent reoperation because of the possible coexistence of invasive carcinoma.

The relatively rare cases of the distant metastasis of DCIS may reflect microvascular invasion into the breast ducts, as the histopathological analysis of surgical specimens has revealed no invasive lesion.^17^, ^31^, ^32^, ^33^ Such metastasis affects breast cancer mortality. The patterns of invasive recurrence of DCIS after treatment include several combinations of local, regional, and distant metastasis.^33^ These findings support the hypothesis that isolated tumor cells in the SLNs and distant metastases in other organs may be simultaneously present at the time of initial diagnosis, and may have developed with breast cancer stem cell activity during disease progression, in some patients with DCIS.^12^ Breast cancer stem cells have been proposed to self‐renew and differentiate to generate non‐neoplastic tumor cells, which form tumor masses and promote tumor progression and metastasis.^34^ Thus, whether systemic therapies other than ET are needed to prevent distant metastasis and improve breast cancer mortality in HR‐positive patients and in HR‐negative or HER‐2‐positive patients with DCIS is unclear. It appears that postoperative RT reduces the local recurrence of non‐invasive carcinoma but does not improve breast cancer mortality.^17^, ^18^, ^19^ Alternatively, the development of invasive carcinoma from non‐invasive carcinoma does not simply increase the distant metastasis rate and breast cancer mortality. Although no evidence of SLN micrometastasis or distant recurrence was observed in this study, probably due to the small number of cases examined, DCIS may inherently have the malignant potential to metastasize to local and distant organs depending on its biological characteristics (e.g., grade, HR status, and HER‐2 expression). Despite the possibility of local control by RT, the reason for the lack of reduction in breast cancer mortality due to the transition of distant metastases has not been elucidated.

Positive surgical margins of ≤2 mm in DCIS resection specimens have been associated with a higher recurrence rate than negative margins, regardless of adjuvant therapy administration.^35^ Two of the three patients with local recurrence in this study had invasive carcinoma that caused local recurrence, although the surgical specimens had negative margins >2 mm. However, both patients had HER‐2‐positive DCIS. HER‐2 overexpression in non‐invasive carcinoma has been reported to contribute to tumor progression and an increased risk of recurrence,^36^ and was likely involved in the development of invasive lesions in these cases. However, the addition of HER‐2 targeting therapy with trastuzumab to RT has been reported to have no significant effect on the local recurrence of HER‐2‐positive DCIS compared with RT alone.^37^ Further studies of the effect of this additional therapy are needed.

The limitations of this study are that it was retrospective and conducted with a small sample. Thus, local recurrence was infrequent, and no distant metastasis was observed in this sample.

## 5 CONCLUSION

Postoperative RT and ET reduce the local recurrence of non‐invasive carcinoma. Local recurrence from non‐invasive to invasive carcinoma does not increase the distant metastasis rate, can be salvaged with additional surgical treatment, and does not affect breast cancer mortality. The clinical significance of the presence of isolated tumor cells in the SLNs with regard to distant metastasis as systemic disease remains to be elucidated. Further studies of isolated tumor cells in SLNs from patients with non‐invasive breast carcinoma will provide a better understanding of tumor progression in these cases.

## AUTHOR CONTRIBUTIONS

**Ryungsa Kim:** Conceptualization (equal); data curation (equal); investigation (equal); methodology (equal); project administration (equal); resources (equal); visualization (equal); writing – original draft (equal); writing – review and editing (equal). **Ami Kawai:** Data curation (equal); formal analysis (equal); investigation (equal); resources (equal); software (equal); visualization (equal); writing – review and editing (equal). **Megumi Wakisaka:** Resources (equal). **Mika Shimoyama:** Resources (equal). **Naomi Yasuda:** Resources (equal). **Mitsuya Ito:** Resources (equal). **Takanori Kin:** Resources (equal). **Koji Arihiro:** Resources (equal).

## FUNDING INFORMATION

No funding was received for this study.

## CONFLICT OF INTEREST

The authors have stated explicitly that there are no conflicts of interest in connection with this article.

## ETHICS STATEMENT

This retrospective cohort study was performed in accordance with the Declaration of Helsinki. The Ethics Committee of Hiroshima Mark Clinic approved the study (no. HMC‐04), and all treatments were administered with the patients' informed consent.

## Data Availability

The datasets used and analyzed during the current study are available from the corresponding author on reasonable request.
